# Predictors of iron consumption for at least 90 days during pregnancy: Findings from National Demographic Health Survey, Pakistan (2017–2018)

**DOI:** 10.1186/s12884-021-03825-2

**Published:** 2021-05-03

**Authors:** Sumera Aziz Ali, Savera Aziz Ali, Shama Razzaq, Nayab Khowaja, Sarah Gutkind, Fazal Ur Raheman, Nadir Suhail

**Affiliations:** 1 Department of Epidemiology, Columbia University, New York, USA; 2 Department of Nursing, University of Alberta, Edmonton, Canada; 3 Department of Community Health Sciences Jinnah Medical and Dental College, Karachi, Pakistan; 4 Liaquat University of Medical and Health Sciences, Jamshoro, Pakistan; 5 Department of Medicine, Aga Khan University, Karachi, Pakistan

**Keywords:** Demographic health survey, Iron consumption, Pakistan, Predictors, Pregnancy

## Abstract

**Background:**

Iron supplementation is considered an imperative strategy for anemia prevention and control during pregnancy in Pakistan. Although there is some evidence on the predictors of iron deficiency anemia among Pakistani women, there is a very limited understanding of factors associated with iron consumption among Pakistani pregnant women. Thus, this study aimed to investigate the predictors of iron consumption for at least ≥90 days during pregnancy in Pakistan.

**Methods:**

We analyzed dataset from the nationally representative Pakistan Demographic Health Survey 2017–2018. The primary outcome of the current study was the consumption of iron supplementation for ≥90 days during the pregnancy of the last birth. Women who had last childbirth 5 years before the survey and who responded to the question of iron intake were included in the final analysis (*n* = 6370). We analyzed the data that accounted for complex sampling design by including clusters, strata, and sampling weights.

**Results:**

Around 30% of the women reported consumed iron tablets for ≥90 days during their last pregnancy. In the multivariable logistic regression analysis, we found that factors such as women’s age (≥ 25 years) (adjusted prevalence ratio (aPR) = 1.52; 95% CI: 1.42–1.62)], wealth index (rich/richest) (aPR = 1.25; [95% CI: 1.18–1.33]), primary education (aPR = 1.33; [95% CI: 1.24–1.43), secondary education (aPR = 1.34; [95% CI: 1.26–1.43), higher education (aPR = 2.13; [95% CI: 1.97–2.30), women’s say in choosing husband (aPR = 1.68; [95% CI: 1.57–1.80]), ≥ five antenatal care visits (aPR =2.65; [95% CI (2.43–2.89]), history of the last Caesarian-section (aPR = 1.29; [95% CI: 1.23–1.36]) were significantly associated with iron consumption for ≥90 days.

**Conclusion:**

These findings demonstrate complex predictors of iron consumption during pregnancy in Pakistan. There is a need to increase the number of ANC visits and the government should take necessary steps to improve access to iron supplements by targeting disadvantaged and vulnerable women who are younger, less educated, poor, and living in rural areas.

## Plain English language summary

Iron deficiency anemia is a major public health problem among women of reproductive age (WRA) mainly in low-middle income countries such as Pakistan. Iron supplementation during pregnancy is considered one of the important strategies to alleviate the burden of anemia in Pakistan. To reduce the prevalence of anemia and associated morbidity and mortality, we need to understand the characteristics of those women most affected by iron deficiency anemia and understand predictors of iron supplement consumption. Although there is some evidence on the predictors of iron deficiency anemia among Pakistani women, there is a dearth of research on the factors associated with iron consumption among women during pregnancy. Thus, we investigated the predictors of consumption of iron supplements for ≥90 days during pregnancy in Pakistan using the nationally representative Demographic Health Survey of Pakistan 2017–2018. The findings of the study revealed that only 30% of the women reported using iron supplements for ≥90 days during pregnancy. Older women with higher education and wealth index, who had a say in choosing their husband, who made at least five antenatal visits during the last pregnancy, and who delivered their baby by Cesarian section were more likely to consume iron supplements for ≥90 days than their counterparts. In the contrast, women from remote regions of Pakistan, such as Baluchistan were less likely to use iron supplements than women from Punjab. The government should take necessary steps to improve access to iron supplements by targeting disadvantageous and vulnerable women living in remote areas of Pakistan with limited access to health care facilities.

## Background

Anemia during pregnancy contributes to a higher burden of morbidity and mortality [1]. The cause of anemia is multifactorial and more than 50% of its burden is attributed to iron deficiency [2]. The existing evidence reveals that approximately one-quarter of reported maternal deaths are due to iron deficiency across the globe [1]. It is estimated that around 120,000 maternal deaths are attributed to severe iron deficiency anemia each year in LMICs [3, 4]. Iron deficiency anemia can lead to adverse perinatal outcomes, such as miscarriage, hemorrhage, macerated and fresh stillbirths, fetal growth retardation, low-birth weight, preterm birth, and neonatal death [5–8]. A meta-analysis revealed that anemic pregnant women are at a higher risk of preterm birth than non-anemic women [9]. Likewise, 44% of preterm births, 25% of babies with low birth weight, and 21% of perinatal mortality are due to anemia during pregnancy in LMICs [10]. Recently, a study based on the World Health Organization (WHO) multi-country survey reported that there is a two-fold increase in risk of maternal mortality due to severe anemia [11]. The findings from a review found that maternal mortality can be plummeted by 29% with each 10 g/L increase in the hemoglobin of women [12].

According to the WHO guidelines, WRA should consume iron supplementation for 3 consecutive months (90 days) in a year to prevent iron deficiency anemia in countries with more than 40% of anemia prevalence [13, 14]. Thus, prescription of iron (tablets or syrup) during pregnancy has become a common practice in most LMICs, however, non-compliance to such guidelines remains an issue [15, 16]. Moreover, women of LMICs tend to consume iron-deficient foods and non-nutritious, less expensive, and easily available foods such as paan, betel nut, and gutka [17, 18]. Therefore, intake of daily oral iron supplements has become a routine practice during pregnancy in most of LMICs to meet requirements of iron for pregnant women [19, 20].

Like other LMICs, anemia is also a serious problem in Pakistan with 41.7 to 77.0% of WRA being affected by anemia in Pakistan as reported by various small studies conducted in different geographic areas of Pakistan [21, 22]. According to a recently conducted nationally representative nutritional survey, 18.2% of WRA are iron deficient in Pakistan, which is more pronounced in rural areas (18.7%) than urban (17.4%) areas [23]. The province of Sindh has the highest proportion of iron deficiency anemia (23.8%) among WRA, followed by Balochistan (19.0%) and Punjab (18.7%) [23]. Anemia can be prevented and controlled effectively by giving iron/folate supplementation to pregnant women in LMICs such as Pakistan [14]. There has been a remarkable improvement in antenatal care (ANC) utilization in Pakistan over the last 5 years, with 86% of pregnant women receiving ANC in 2017 compared with only 55% in 2012 [24]. Even though iron supplementation is an essential part of ANC, 58.9% of pregnant women receive iron supplements during their ANC visits and only 29% use iron supplements for ≥90 days [24]. Moreover, proportion of women using iron or folic acid supplements during pregnancy is even lower in Pakistan compared with other countries in South Asia [25–27]. The evidence suggests that if we could decrease the burden of iron deficiency anemia by providing iron supplementation to women, we could avert around 3190 disability-adjusted life years (DALYs) in the short and long term [28]. To do that we need to understand the characteristics of those most affected by iron deficiency anemia and understand predictors of iron supplement use. Although there is some evidence on the predictors of iron deficiency anemia among Pakistani women [21, 22, 29], there is a very limited understanding of the factors associated with iron consumption among women, in general, and during pregnancy, in particular, due to a dearth of studies in this area at the national level. Despite the evidence that anemia can effectively be addressed by the consumption of iron supplements by pregnant women, none of the studies in Pakistan have assessed the factors associated with iron consumption during pregnancy. Hence, there is a need to investigate the underlying predictors of iron consumption comprehensively in Pakistan. Understanding these predictors holistically will enable the development of local strategies and targeted interventions to enhance the uptake of iron by pregnant women. Thus, the current study aimed to investigate the predictors of consumption of iron supplements for ≥90 days during pregnancy in Pakistan. Additionally, we hypothesized that predictors of consumption of iron may vary across place of residence, therefore, we studied these predictors across rural and urban areas.

## Methods

### Study design and setting

The Pakistan Demographic Health Survey (PDHS) 2017–18 is the fourth national cross-sectional survey in the Demographic Health Survey International Series and follows surveys in 1990–91, 2006–07, and 2012–13 [24]. The data was collected across the country from November 2017 to April 2018. High-quality data were collected on different domains such as maternal and child health, family planning, maternal nutrition, and other important health-related issues [24]. We performed an analysis based on the national level available data to investigate the predictors of iron consumption among pregnant women.

### Study sample and sampling technique

A stratified two-stage sample design was followed in the 2017–18 PDHS. The sampling frame was based on the record of enumeration blocks (EBs) generated for the Pakistan Census 2017. The 2017–18 PDHS represents the population of all four provinces of Pakistan including Punjab, Sindh, Baluchistan, Khyber Pakhtunkhwa (KPK), and other regions such as Azad Jammu and Kashmir (AJK), Gilgit Baltistan (GB), Islamabad capital territory (ICT), and the former Federally Administrated Tribal Areas (FATA) [24]. Using a stratified two-stage sample design, eight regions were stratified into urban and rural areas thus resulting in 16 sampling strata. A two-stage selection process was used in every stratum to select an independent sample. Clusters made of EBs were selected as the first sample points. Probability proportional to their size (number of households in each EB) was implemented to use the EBs during the survey. A total of 580 clusters were selected. This was followed by systematic sampling of households during the second stage of sampling. A household listing process was conducted in the identified clusters, and around 28 households were selected per cluster with an equal probability of systematic selection process thus resulting in 16,240 households [24].

### The study population for the analysis

The 2017–18 PDHS included all women of reproductive age who were married at the time of the survey. The eligibility for the interview was contingent on being a permanent resident of the identified households or a visitor who came to stay in the chosen households 5 years before the survey. Based on the eligibility criteria, around 12,364 WRA were interviewed during the 2017–18 PDHS survey across the country. However, for the current analysis, we limited our sample to women who had childbirth 5 years preceding the survey, had responded to the question of iron intake during their last pregnancy, and who had complete data  on important variables such as husband’s education, consumption of tobacco, and women’s say in choosing a husband. Thus, we were able to include 6370 women in the final analysis.

### Outcome variable

The outcome variable was the consumption of iron supplementation for ≥90 days during the pregnancy of the last birth. We used two questions from PDHS-2017 to 2018 to assess if women had utilized iron for ≥90 days during the pregnancy of their last birth. These questions were: “During this pregnancy, were you given, or did you buy any iron tablets or iron syrup? During the whole pregnancy, for how many days did you take the tablets or syrup?”

### Study variables

We selected the study variables after conducting a detailed literature review. We considered those variables for the analysis that are associated with the iron consumption in the literature and were also available in the PDHS 2017–2018 data set with complete information. We categorized these variables into five major types : socio-demographic predictors [(mother’s age (in years), women’s and her husband’s education (no education, primary, secondary, and higher education), maternal working status (Yes/No), women’s say in choosing husband (Yes/No), wealth index (Poor, middle and rich)]; reproductive factors [(parity, number of children ever born), births in the last 5 years, ANC visits (number of visits during last pregnancy), and mode of the last delivery (vaginal or caesarian-section)]; body mass index (BMI) measured in kg/m^2^; behavioral factors [(currently smoking cigarettes (Yes/No), chewing tobacco (Yes/No)]; and contextual factors [(place of residence (urban/ rural)) and geographical regions (Punjab, Sindh, KPK, Baluchistan, ICT, AJK, and GB)].

### Statistical analysis

We performed descriptive statistics and regressions to analyze the data. We adjusted all the descriptive and regression analysis models after considering the complex sampling design of the PDHS 2017–2018. We used a complex sample analysis procedure by considering the primary sampling units (PSUs) as clusters, strata, and sampling weights in the models to account for the cluster sampling design to provide precise estimates of frequency, proportion, and prevalence ratios [30, 31]. We generated frequencies and percentages of independent and dependent variables. We used the chi-square test (χ2) to study the association between the categorical independent variables of interest with the binary outcome variable. We used the log-binomial model (surveygenmod with a log-link) to create prevalence ratios for univariable and multivariable analysis. We performed a univariable analysis to examine the predictors of utilizing iron for ≥90 days during pregnancy of the last birth. Additionally, we performed multivariable regression using variables that were significant with an alpha of 0.05 level in the univariable analysis. We constructed three models to assess factors at different levels. The reason for model 0 was to assess one-to-one relationship (unadjusted) between one individual-level factors and outcome without adjusting the other contextual or individual-level factors. This model is important to assess that how much a relationship between a variable of interest and outcome changes after adding other confounders that could confound the relationship. Therefore, a baseline model is necessary to compare the unadjusted results with the adjusted ones. Hence, model 0 generated the results of the univariable association between the outcome and all independent study variables. Model 1 is the adjusted model and the rationale for this model is to see how the relationship between independent variable and outcome is confounded by individual-level factors associated with mother. We only included factors that are directly related to the woman herself. Model 1 was adjusted for individual-level characteristics such as women’s age, mother’s education level, women’s say in husband’s choice, wealth index, ANC visits, and mode of delivery. Therefore, we included other factors such as the husband’s education and region in a separate model. Model 2 provided results after controlling for variables that were not directly related to women such as husband’s education and region. Finally, we performed domain analysis (sub-group) by considering place of residence as a domain or stratum and presented results separately for rural and urban areas of Pakistan. This sub-group analysis was done to assess whether predictors of iron consumption vary across the place of residence (rural/urban). Because our outcome of interest was common (29% of women consumed iron supplements for ≥90 days), we reported crude and adjusted prevalence ratios (aPR) and respective 95% confidence intervals (CI) after adjustment for strata and clustering at PSU level. We considered *P*-values of < 0.05 as statistically significant. We used the software SAS 9.4 for data analysis.

## Results

### Descriptive characteristics of study participants

We found that around 29.68% of the women reported consuming iron for ≥90 days during their last pregnancy. About a quarter of women (23.26%) were younger than 25 years old and 59.15% had more than two children (Table 1). Moreover, 48.01% of women and 28.94% of their husbands were not educated. Around half of the women (51.86%) were poor or poorest, 82.13% reported not working after marriage, and a similar proportion (80.95%) had their say in choosing the husband. Approximately one-third of women (37.69%) had made ≥ five ANC visits during their last pregnancy and 23.81% delivered their baby via caesarian-section. Around two-thirds of the study participants (66.81%) were living in rural areas and around half of them (51.07%) were from Punjab province and approximately one-quarter (24.06%) were from Sindh province (Table 1).

**Table 1:** Socio demographic and reproductive characteristics women of reproductive age who reported using iron tablets or syrup during pregnancy of their last birth (*n* = 6370)

Characteristics			Use of iron (tablets/syrup) during last pregnancy			
			< 90 days (***n*** = 4497)		≥ 90 days (***n*** = 1891)	
	n	Proportion	n	%	n	%
**Women’s age**						
< 25 years	1522	23.26	1127	25.0	395	19.0
≥ 25 years	4848	76.74	3352	75.0	1496	81.0
**Parity**						
≤ 2 children	2546	40.85	1684	40.0	862	44.0
> 2 Children	3824	59.15	2795	60.0	1029	56.0
**Births in last 5 years**						
≤ 1 birth	3475	53.67	2369	52.0	1106	58.0
> 1 birth	2895	46.33	2110	48.0	785	42.0
**Women’s education**						
No education	3435	48.03	2795	55.0	640	31.0
Primary	854	16.34	590	17.0	264	16.0
Secondary	1219	22.19	741	20.0	478	28.0
Higher	862	13.44	353	8.0	509	25.0
**Wealth Index**						
Poor/Poorest	2756	51.86	2294	48.0	462	26.0
Middle	1202	25.27	856	22.0	334	18.0
Rich/Richest	1222	22.86	1329	30.0	1095	57.0
**Worked after marriage**						
No	5454	82.13	3832	81.0	1622	84.0
Yes	916	17.87	647	19.0	269	16.0
**BMI (Kg/m2)**^a^						
< 18.5 kg/m2	211	10.46	164	4.0	47	30
18.5–24.9 kg/m2	1020	44.15	740	17.0	280	15.0
≥ 25 kg/m2	1151	45.39	748	15.0	403	21.0
**Antenatal care visits**						
< 5 visits	4057	62.31	3368	73.0	689	38.0
≥ 5 visits	2313	37.69	1111	27.0	1202	62.0
**Last Caesarian-section**						
No	5135	76.19	3844	81.0	1291	65.0
Yes	1235	23.81	635	19.0	600	35.0
**Say in husband’s choice**						
No	1352	19.05	1066	22.0	286	13.0
Yes	5018	80.95	3413	78.0	1605	87.0
**Chew tobacco**						
No	6272	98.85	4400	99.0	1872	99.0
Yes	98	1.15	79	1.0	19	1.0
**Husband’s education**						
No education	1872	28.94	1574	34.0	298	18.0
Primary	907	16.41	677	18.0	230	13.0
Secondary	2148	35.26	1479	35.0	669	36.0
Higher	1443	19.39	749	14.0	694	33.0
**Place of residence**						
Rural	3427	66.81	2632	71.0	795	57.0
Urban	2943	33.19	1847	29.0	1096	43.0
**Region**						
Punjab	1628	51.07	1110	50.0	518	53.0
Sindh	1417	24.06	950	23.0	467	27.0
KPK	1307	16.42	920	17.0	387	15.0
Balochistan	885	5.28	762	6.0	123	3.0
FATA	637	2.42	499	3.0	138	2.0
ICT	496	0.76	238	1.0	258	1.0

^a^BMI data were only collected from one third of the sample thus data was missing for 3988 women

### Characteristics of study participants by consumption of iron during the last pregnancy

A significantly higher percentage of the women consuming iron less than 90 days (25.0%) were younger than 25 years old when compared with 19.0% of the women consuming iron for ≥90 days (*p*-value: < 0.001). Similarly, 44% of the women consuming iron for ≥90 days and 40% of their counterparts had at most two children (p-value: < 0.001). A significantly lower proportion of women consuming iron for ≥90 days (31.0%) were not educated compared with their counterparts (55.0%; p-value: < 0.001). About one-quarter of the women (26.0%) consuming iron for ≥90 days were poor/poorest when compared with 48% of women consuming iron for less than 90 days (Table 1). Around three fourth of women consuming iron for ≥90 days (62.0%) had made ANC visits during their last pregnancy as opposed to 27.0% of the women consuming iron for less than 90 days. Around 43.0% of the women consuming iron for ≥90 days were from urban areas as opposed to 29.0% of the women consuming iron for less than 90 days. However, there were no significant differences between women consuming iron for ≥90 days and their counterparts by working status, using cigarettes, chewing tobacco, type of relationship with husband, and history of termination of pregnancy.

### Predictors of iron consumption for ≥90 days during the last pregnancy: findings of bivariate analysis

It was found that ≥25 years old women were 30% (PR = 1.30; [95% CI: 1.15–1.47]) more likely to use the iron for ≥90 days when compared to less than 25 years old women (Table 3). Likewise, women with higher education were 2.89 times likely to use the iron for ≥90 days when compared to the uneducated women (PR = 2.89; [95% CI:2.48–3.38]). Similarly, rich, or richest women were 2.39 times likely to use the iron for ≥90 days when compared to their counterparts (PR = 2.39; [95% CI:1.98–2.88]). Furthermore, women who had made ≥5 ANC visits, were 2.69 (PR = 2.69; [95% CI: 2.40–3.01]) times likely to use the iron for ≥90 days when compared with women who made less than 5 ANC visits. Women who had a say in choosing their husband and who delivered their last baby by Caesarian-section were 1.58 (PR = 1.58; [95% CI: 1.36–1.85]) and 1.74 (PR = 1.74; [95% CI: 1.56–1.94]) times as likely to use the iron for ≥90 days respectively when compared to their counterparts (Table 3). Women who were living in Baluchistan and FATA were 53% (PR = 0.47; [95% CI: 0.36–0.62]) and 37% (PR = 0.63; [95% CI: 0.42–0.96]) less likely to use iron for ≥90 days, respectively, when compared to women living in Punjab as shown in Table 2.

**Table 2:** Predictors of iron consumption for ≥90 days during pregnancy among women in Pakistan (*n* = 6370)

				Model 1			Model 2		
	Crude Estimates			Adjusted Estimates			Adjusted Estimates		
Variables	PR	95% CI		PR	95% CI		PR	95% CI	
**Individual level factors**									
**Women’s age**									
< 25 years	1			1			1		
≥ 25 years	1.30	1.15	1.47	1.98	1.74	2.25	1.52	1.42	1.62
**Women’s education**									
No education	1			1			1		
Primary	1.49	1.25	1.78	1.00	0.89	1.14	1.33	1.24	1.43
Secondary	1.97	1.67	2.32	0.88	0.78	1.00	1.34	1.26	1.43
Higher	2.89	2.48	3.38	3.16	2.72	3.68	2.13	1.97	2.30
**Say in Husband’s choice**									
No	1			1			1		
Yes	1.58	1.36	1.85	1.96	1.74	2.21	1.68	1.57	1.80
**Wealth Index**									
Poor/Poorest	1			1			1		
Middle	1.41	1.15	1.74	0.62	0.50	0.76	0.94	0.87	1.00
Rich/Richest	2.39	1.98	2.88	1.21	1.03	1.40	1.25	1.18	1.32
**Antenatal care visits**									
< 5 visits	1						1		
≥ 5 visits	2.69	2.40	3.01	3.18	2.75	3.66	2.65	2.43	2.89
**Last Caesarian-section**									
No	1			1			1		
Yes	1.74	1.56	1.94	1.45	1.34	1.57	1.29	1.23	1.36
**Other Factors**									
**Regions**									
Punjab	1				NA		1		
Sindh	1.07	0.89	1.29				1.33	1.26	1.40
Balochistan	0.47	0.36	0.62				0.77	0.73	0.81
KPK	0.86	0.70	1.06				1.36	1.27	1.46
ICT	1.70	1.42	2.02				2.09	1.93	2.25
FATA	0.63	0.42	0.96				1.29	1.08	1.54
**Husband’s education**									
No education	1				NA		1		
Primary	1.30	1.04	1.63				0.95	0.85	1.06
Secondary	1.65	1.39	1.94				0.95	0.85	1.06
Higher	2.74	2.33	3.23				1.99	1.83	2.17

Model 1: Age, women’s education, wealth index, had say in choosing husband, ANC visits, last Caesarian-sections

Model 2: All variables in Model 1 plus region and husband’s education

*PR* prevalence ratio, *NA* not applicable

### Predictors of iron consumption for ≥90 days during the last pregnancy: findings of multivariable analysis

The results of the multivariable analysis for Model 1 were adjusted for women’s age, education, wealth index, her say in husband’s choice, number of ANC visits, and the last Caesarian-section. Model 2 adjusted results for all variables in Model 1 in addition to region and husband’s education. The results of Model 1 demonstrated that associations between women’s age (aPR = 1.98; [95% CI: 1.74–2.25]), higher level of women’s education (aPR = 3.16; [95% CI: 2.72–3.68]), women’s say in choosing husband (aPR = 1.96; [95% CI: 1.74–2.21]), ≥ five ANC visits (aPR = 3.18; [95% CI (2.75–3.66]) and iron consumption for ≥90 days persisted in the final adjusted model. Indeed, associations became stronger in the adjusted model. However, the association between wealth index (rich/richest) (aPR = 1.21; [95% CI: 1.03–1.40]), last Caesarian-section (aPR = 1.45; [95% CI: 1.34–1.57]) and iron consumption for ≥90 days also persisted in the adjusted model but became weaker relative to the bivariate analysis as shown in Table 2.

The findings from Model 2 demonstrated that associations between women’s age (aPR = 1.52; 95% CI: 1.42–1.62)], women’s say in choosing husband (aPR = 1.68; [95% CI: 1.57–1.80]), ≥ five ANC visits (aPR =2.65; [95% CI (2.43–2.89]), last Caesarian-section (aPR = 1.29; [95% CI: 1.23–1.36]) and iron consumption for ≥90 days persisted in the Model 2, but became weaker relative to Model 1. However, the association between wealth index (rich/richest) (aPR = 1.25; [95% CI: 1.18–1.32]), women’s primary (aPR = 1.33; [95% CI: 1.24–1.43) and secondary education (aPR = 1.34; [95% CI: 1.26–1.43) and iron consumption for ≥90 days became more stronger as opposed to Model 1 as shown in Table 3. The Model 2 revealed that women from Sindh (aPR = 1.33; [95% CI: 1.26–1.40), KPK (aPR = 1.36; [95% CI: 1.27–1.46]), ICT (aPR = 2.09; [95% CI: 1.93–2.25]), and FATA (aPR = 1.29; [95% CI: 1.08–1.54]) were more likely to consume iron supplement when compared with women from Punjab. However, women from Baluchistan were 23% (PR = 0.77; [95% CI: 0.73–0.81]) less likely to consume iron for ≥90 days when compared to women living in Punjab. Besides, women of highly educated husbands were 1.99 times likely (aPR = 1.99; [95% CI: 1.83–2.17]) to use iron for ≥90 days when compared to their counterparts as shown in Table 2.

**Table 3:** Predictors of iron consumption for ≥90 days during pregnancy among women in Pakistan by place of residence (*n* = 6370)

	Urban			Rural		
	Adjusted Estimate			Adjusted Estimate		
Variables	PR	95% CI		PR	95% CI	
**Woman’s age**						
< 25 years	1			1		
≥ 25 years	1.22	0.90	1.67	1.98	1.74	2.25
**Women’s education**						
No education	1			1		
Primary	0.99	0.65	1.53	1.00	0.89	1.14
Secondary	1.26	0.84	1.87	0.88	0.78	1.00
Higher	2.26	1.41	3.64	3.16	2.72	3.68
**Say in Husband’s choice**						
No	1					
Yes	1.20	0.88	1.65	1.96	1.74	2.21
**Wealth Index**						
Poor/Poorest	1			1		
Middle	0.87	0.47	1.60	0.62	0.50	0.76
Rich/Richest	1.19	0.66	2.16	1.21	1.03	1.40
**Antenatal care visits**						
< 5 visits	1			1		
≥ 5 visits	2.59	1.94	3.48	3.28	2.51	4.29
**Last Caesarian-section**						
No	1					
Yes	1.18	0.94	1.50	1.45	1.34	1.57

### Predictors of iron consumption for ≥90 days during the last pregnancy: findings of domain or sub-group analysis

Sub-population analysis by place of residence showed that in rural areas, older women (≥ 25 years) (aPR = 1.98; [95% CI: 1.74–2.25]), who had say in choosing their husband (aPR = 1.96; [95% CI: 1.74–2.21]), rich/richest women (aPR = 1.21; [95% CI: 1.03–1.40]), and who delivered their last baby by Caesarian-section (aPR = 1.45; [95% CI: 1.34–1.57]) were more likely to use iron when compared with their counterparts (Table 3). However, the results for the same predictors were not statistically significant for women residing in urban areas as shown in Table 3. On the other hand, women with higher education were more likely to use the iron for ≥90 days than women with no education both in urban (aPR = 2.26; [95% CI: 1.41–3.64]) and rural areas (aPR = 3.16; [95% CI: 2.72–3.68]). Likewise, women who made ≥5 ANC visits were more likely to use the iron for ≥90 days when compared with women who made less than 5 ANC visits both in urban (aPR = 2.59; [95% CI: 1.94–3.48]) and rural areas (aPR = 3.28; [95% CI: 2.51–4.29]), however, the magnitude of the estimate was relatively higher for women in rural areas as opposed to urban women as shown in Table 3.

## Discussion

This is one of the first studies that investigated the predictors of iron consumption during pregnancy among women of reproductive age in Pakistan. Our study reported that more than a quarter of women reported consuming iron for ≥90 days. Factors such as women’s age, education, wealth index, say in choosing husband, husband’s education, ANC visits, history of the last caesarian-section, and region of the country were found to be important predictors of iron consumption for ≥90 days among Pakistani women. The results on the predictors of iron consumption for ≥90 days among Pakistani women should be interpreted in the economic and socio-cultural context of Pakistan.

Our study outcome regarding the proportion of women consuming iron for ≥90 days is not comparable with the other studies conducted in LMICs such as Malawi or Ethiopia, where the prevalence of women consuming iron for ≥90 days has been reported to be 37 and 3.5% respectively [32, 33]. On the contrary, iron utilization during pregnancy in LMICs such as the Philippines and Senegal has been reported as 70 and 69% respectively, which is relatively higher than other LMICs [34, 35]. Generally, it seems that the proportion of women consuming iron is lower in developing countries including Pakistan as opposed to developed countries. For instance, iron consumption by pregnant women in the USA and the Netherlands is 72 and 77%, respectively [36, 37]. It is crucial to note that a direct comparison of our findings with studies conducted in other countries may not be possible due to differences in the demographics of the study participants included in the survey, the number of days for iron consumption, study settings, health-seeking behavior, and socio-cultural circumstances.

The findings regarding the association of women’s age with the consumption of iron are comparable to the other studies such as Tanzania and Sudan where older women have been reported to consume the iron for the recommended number of days than younger women [38–41]. Overall, these findings can be explained by the fact that older women might be more knowledgeable, experienced, and health-conscious than younger women, thus, tend to use iron more than younger women. Moreover, it might be possible that older-aged women in Pakistan might be more experienced and knowledgeable for pregnancy and childbirth-related outcomes, thus, might feel the need to take iron supplements more than younger and less experienced women. However, our findings contradict the study conducted in Malawi, where younger women were found to consume iron as recommended when compared with older women [32]. These differences in the findings may be due to differences in the health-seeking behavior and experience of women of different ages in the two different countries.

A positive association was found between women’s education, increased wealth index, and iron consumption in Pakistani women, and this finding is confirmed in several studies conducted in few districts of Pakistan, Malawi, Ethiopia, and India where women with higher education and more wealth are more likely to consume iron than less-privileged women [32, 33, 42, 43]. Collectively, these findings can be elucidated by the fact that educated and wealthy women might be more knowledgeable and have greater access to information pertinent to iron deficiency anemia and its associated preventive and control measures, such as intake of iron supplementation particularly during pregnancy. Besides, educated women are usually more concerned about their upcoming baby’s health when compared with non-educated women [44]. Likewise, it is more likely that rich women with more wealth have high purchasing power and access to resources than poor women. These findings regarding higher wealth index and utilizing iron during pregnancy are also confirmed by the previously conducted demographic surveys in Pakistan, Bangladesh [27], India [25], and Nepal [26], where rich women with higher education are reported to consume iron supplements for a prolonged duration.

As expected, we found a positive association between the number of ANC visits and iron consumption for ≥90 days. In Pakistan, ANC is considered one of the important routes to deliver iron supplements to pregnant women and this is also true for many other developing countries [45–47]. For example, a mixed-method study conducted in Cambodia found ANC as the only way to take iron supplements during pregnancy [48]. Our findings regarding positive association between ANC visits and iron consumption are analogous with findings from other studies such as Malawi and Vietnam where women with more ANC visits have been found to consume iron for the recommended number of days during pregnancy [32, 49]. However, one of the studies conducted in Tanzania found an inverse association between number of ANC visits and iron consumption. These contradictory findings may be due to the differences in the type of services being provided to pregnant women during ANC. Unlike Pakistan where women are provided free of cost iron supplements, women in Tanzania are only counseled for the intake of iron during ANC visits [38]. Moreover, women in Tanzania have to pay co-payment to take iron supplements, which might prevent women from purchasing iron tablets despite making sufficient ANC visits [38].

Our findings revealed that women who delivered by caesarian-section were more likely to consume iron for ≥90 days than their counterparts. Women with planned or elective caesarian-sections might have been counseled by their health care providers to consume iron regularly with recommended dosage and duration to avoid unnecessary blood transfusions at the time of delivery. For example, according to the National Health Service Blood transfusion guidelines, women with planned Caesarian-sections should be screened for anemia and should be advised to take iron supplements either orally or parenterally depending upon the severity of anemia [50, 51]. In addition, our study findings revealed that women who had a say in choosing their husbands were more likely to consume the iron for ≥90 days when compared to those who did not have a say in choosing their husbands. Having one’s say in choosing the husband in the Pakistani context indirectly alludes to the autonomy or empowerment of the women. Thus, it might be possible that such women might be more empowered, thus consuming iron supplements for the required number of days when compared with the women who did not have a say in choosing their husbands. There is a evidence that autonomous women are more likely to make ANC visits; thus, tend to consume iron more than non-autonomous women [52, 53]. Moreover, these women might have freedom of mobility, and control over financial resources to access the services they need [54].

Lastly, women from Baluchistan province were less likely to consume iron for ≥90 days when compared to women from Punjab province. This finding could be justified that Baluchistan is a remote and underdeveloped province of Pakistan with limited access to health care facilities [55]. Women’s access to health services is very limited due to the scattered population and the absence of healthcare infrastructure [56]. A vast majority of women are uneducated, and their mobility is limited mainly in tribal areas of Baluchistan [57]. Moreover, only 50% of the women utilize ANC services during their pregnancy, which is the lowest percentage when compared to the other regions of the country [24]. Given these factors and based on the findings of our study, the government of Pakistan must take necessary steps to improve access to maternal health services during pregnancy in general with improved access to iron supplements in particular to reduce the burden of anemia in the region.

### Strengths and limitations

This is one of the first studies in Pakistan that investigated predictors of iron consumption for ≥90 days during pregnancy at the national level. Larger samples of women selected through appropriate sampling technique provided a representative sample of women, thus findings can be generalized to the whole country and neighboring countries with similar socio-demographic characteristics. Moreover, during the actual survey, the investigators trained the interviewers to ask questions from respondents in their local languages across different regions of the country to collect correct information. Unlike previous studies, we generated prevalence ratios to avoid overestimating the effects of true estimates. However, our study findings need to be interpreted considering some inherent caveats. First, the survey does not allow temporal precedence to be unambiguously determined, thus, limiting to conclude causality of the predictors studied for the consumption of iron for ≥90 days during pregnancy. Second, there could be a possibility of recall bias as women were asked about the consumption of iron intake during the pregnancy of the last birth. This was further augmented by self-reporting method to measure the outcome of the study thus might underestimate or overestimate the consumption of iron during pregnancy. There is evidence that self-reporting usually overestimates the actual compliance as opposed to counting the pills by the researcher or measuring the compliance by some biochemical markers [34, 58].

## Conclusions

Our study confirmed that slightly more than a quarter of women consumed iron for ≥90 days. Women’s age, education and spouse education, number of antenatal care visits, the region of the country, woman’s say in choosing her husband, husbands’ education, and history of the last caesarian-sections were found to be important predictors of iron consumption for ≥90 days during the pregnancy in Pakistan.

### Implications and future directions

There could be important policy implications in the future. The government should take necessary steps to improve access to iron supplements to all women during pregnancy. More specifically, the government should target disadvantageous and vulnerable women who are younger, less educated, poor with less purchasing power, living in remote areas of Pakistan, and with limited access to health care facilities. We also suggest few programmatic aspects such as Pakistan’s Lady Health Workers program can play a vital role to provide supplements to pregnant women at their doorsteps who cannot make the required number of ANC visits to take their iron supplements. There is a need to improve access to women living in remote and far-flung areas who cannot visit health care facilities easily. In addition, there is a need to design combined nutrition and educational strategies to encourage rural women to consume iron-rich foods in their diet in addition to consuming the recommended dosage of iron supplements during pregnancy. Proactive efforts can be made to educate women through effective campaigns with the support of Lady Health Workers program for motivating them to take recommended iron tablets.

Not Applicable.

## Data Availability

Data is publicly accessible at the DHS website.

## Abbreviations

ANC: Antenatal care
AJK: Azad Jammu and Kashmir
CI: Confidence interval
DALYs: Disability-adjusted life years
GB: Gilgit Baltistan
EBs: Enumeration blocks
ERC: Ethical review committee
FATA: Federally administrated tribal areas
ICT: Islamabad capital territory
KPK: Khyber Pakhtunkhwa
LMICs: Low Middle Income Countries
PDHS: Pakistan Demographic Health Survey
PR: Prevalence ratio
PSUs: Primary sampling units
WHO: World Health Organization
WRA: Women of Reproductive Age
